# Evaluation of Transplacental Antibody Transfer in Pregnant Women Immunized with Different SARS-CoV-2 Homologous or Heterologous Schemes

**DOI:** 10.3390/vaccines11020415

**Published:** 2023-02-11

**Authors:** Maria Elena Romero-Ibarguengoitia, Zulema Lourdes Flores-Salazar, Kimberly Dariela Arroyo-García, Rafael Soto-Gámez, Jessica Andrea Leal-Meléndez, Mauricio René Garza-Herrera, Gordon Bennett-Vidales, Mauricio Hurtado Cabrera, Roberto González-Habib, Liliann Peña Jiménez, Raúl Garza-Bulnes, Irene Antonieta Barco-Flores, Luis Fernando Castillo-Figueroa, Arnulfo Garza-Silva, Andrea Rivera-Cavazos, Diego Rivera-Salinas, Arnulfo González-Cantú, Miguel Ángel Sanz-Sánchez

**Affiliations:** 1 Research Department, Hospital Clínica Nova de Monterrey, San Nicolás de los Garza 66450, Nuevo León, Mexico; 2 Especialidades Médicas, Escuela de Medicina, Universidad de Monterrey, Avenida Morones Prieto, 4500-Pte, Zona Valle Poniente, San Pedro Garza García 66238, Nuevo León, Mexico; 3 Dirección de Enseñanza e Investigación en Salud, Hospital Christus Muguerza Sistema de Salud, Calle Miguel Hidalgo y Costilla 2525, Obispado, Monterrey 64000, Nuevo León, Mexico; 4 Departamento de Neonatología, Hospital Christus Muguerza Conchita, Calle 15 de Mayo 1822, María Luisa, Nuevo Obispado, Monterrey 64040, Nuevo León, Mexico; 5 Departamento de Ginecología, Hospital Christus Muguerza Conchita, Calle 15 de Mayo 1822, María Luisa, Nuevo Obispado, Monterrey 64040, Nuevo León, Mexico; 6 Departamento de Ginecología, Hospital Clínica Nova de Monterrey, Avenida del Bosque 139, Cuauhtémoc, Cuauhtémoc, San Nicolás de los Garza 66450, Nuevo León, Mexico; 7 Dirección de Enseñanza e Investigación en Salud, Hospital Christus Muguerza Conchita, Calle 15 de Mayo 1822, María Luisa, Nuevo Obispado, Monterrey 64040, Nuevo León, Mexico; 8 Departamento de Neonatología, Hospital Clínica Nova de Monterrey, Avenida del Bosque 139, Cuauhtémoc, Cuauhtémoc, San Nicolás de los Garza 66450, Nuevo León, Mexico

**Keywords:** COVID-19, vaccines, passive antibody transfer, newborn

## Abstract

There is scarce information related to transplacental antibody transfer against severe acute respiratory syndrome coronavirus 2 (SARS-CoV-2) with different homologous and heterologous vaccination schemes. This study aimed to correlate the magnitude of transplacental transfer anti-SARS-CoV-2 antibodies in different homologous and heterologous schemes. An observational cross-sectional study was developed to identify pregnant women vaccinated against SARS-CoV-2. They were questioned about their immunization status; blood samples from the mother, umbilical cord during labor, and the newborn 72 h after birth were taken to measure anti-S1 and anti-S2 specific IgG antibodies for SARS-CoV-2. We recruited 104 women with a median age of 29 (SD 1.17). We found antibodies in all newborns with vaccinated mothers. Homologous BNT162b2 mRNA regimen had the highest mean (SD) antibody titers (AU/mL) in maternal (994.93 (3.08), *p* = 0.039), umbilical cord (1316.43 (2.79), *p* = 0.016), and newborn (1192.02 (3.55), *p* = 0.020) blood. The generalized linear model showed a positive effect over antibodies with at least one dose in maternal (β = −1.1, *p* = 0.002) and newborn (β= −0.717, *p* = 0.044) blood, and with two doses (β = −0.684, *p* = 0.026) in umbilical cord blood. In conclusion, antibodies were detected in all vaccinated women and their newborns. Transfer of antibodies was found from the first dose, and the levels increased with the number of vaccine doses. Vaccination should be encouraged in pregnant women with any available scheme.

## 1. Introduction

Severe acute respiratory syndrome coronavirus 2 (SARS-CoV-2) infection has been a public health issue since 2019 due to emerging cases of atypical pneumonia [1,2,3]. The World Health Organization (WHO) declared this situation as the COVID-19 pandemic in March 2020 because of the worldwide spread of the virus with high mortality [4,5,6]. SARS-CoV-2 is contagious to anyone exposed to the virus; however, children and pregnant women are at higher risk. Severe COVID-19 during pregnancy has been associated with a higher risk of preterm birth and its related comorbidities [7,8,9,10,11]. Newborns have an immature immune system, relying only on transplacental passive immunization where IgG antibodies reach fetal circulation [12,13,14].

Vaccination against SARS-CoV-2 has become a top-priority research field due to the lack of treatments for the disease and the need to decrease the number of cases and mortality. According to the WHO, there are around 200 clinical trials in different phases for SARS-CoV-2 vaccines [15]. These vaccines can be divided depending on their mechanism of action, all of which lead to an immune response against SARS-CoV-2. In mRNA vaccines, such as BNT162b2 mRNA and mRNA-1273, modified RNA produces an immune response against SAR-CoV-2’s spike proteins. Viral vector vaccines (ChAdOx1-S, Ad26.COV2.S, Ad5-nCoV, and Gam-COVID-Vac) use a genetically changed virus. Inactivated virus vaccines, such as Coronavac, use a weakened virus incapable of producing disease but with immunogenicity. In protein subunit vaccines, such as NVX-CoV2373, protein fragments or shells mimic SARS-CoV-2 and are capable of producing an immune response [16].

Pregnant women and pediatric patients were initially excluded from pre-authorization clinical trials [17,18]. However, the American College of Obstetricians and Gynecologists and the American Academy of Pediatrics recommend vaccination for everyone over 6 months of age [19,20,21,22]. In Mexico, vaccination for pregnant women was authorized with BNT162b2 mRNA, ChAdOx1-S, Ad5-nCoV, Gam-COVID-Vac, Coronavac, or Ad26.COV2 in May 2021. The authorization and availability of different types of vaccines for pregnant women in Mexico provide researchers the possibility to study these vaccines’ reactogenicity, efficacy, immunogenicity, and after-birth seroconversion of the fetus, leading to a better comprehension of the SARS-CoV-2 vaccines and their benefits in pregnant women and newborns [23].

Few studies involve vaccination against SARS-CoV-2 during pregnancy and the transplacental transfer of IgG antibodies to newborns. Studies have been conducted involving only mRNA vaccines, such as BNT162b2 mRNA and mRNA-127324-29 [24,25,26]. There are no studies involving other types of vaccines and heterologous vaccination schemes in pregnant women. Therefore, the objective of this study was to evaluate the transfer of anti-S1/S2 antibodies against SARS-CoV-2 to newborns from pregnant women that received different vaccination schemes (homologous and heterologous).

## 2. Materials and Methods

This observational, cross-sectional, descriptive, comparative study was conducted between April 2022 and October 2022. Participants were pregnant women that had received at least one of the vaccines against SARS-CoV-2 authorized by the Mexican Health System, which were BNT162b2, ChAdOx1-S, Ad5-nCoV, mRNA-1273, Gam-COVID-Vac CoronaVac, or Ad26.COV2. For the development of the study, the STROBE reporting guidelines were followed [24]. The local Institutional Review Board approved the study (Ref.:18102021-CN-PED-CI, dated 28 February 2022) conducted according to The Code of Ethics of the World Medical Association (Declaration of Helsinki) for human experiments. Due to this study’s nature, it was necessary to obtain a consent form.

The inclusion criteria were pregnant women who had received at least one dose of the vaccine against SARS-CoV-2, who were receiving active medical service at HCN (Hospital Clinica NOVA, a private hospital in Northern Mexico), who had given birth at CMCH (Christus Muguerza Conchita Hospital, a private hospital in Northern Mexico), and who agreed to sign a consent form to participate. The exclusion criteria were newborns who presented hemodynamic or ventilatory instability, patients who refused to allow samples to be taken from them, and patients who had a problem with the collection, storage, or processing of the sample.

All women with a vaccination history for COVID-19 who agreed to participate in the study and who belonged to the HCN health service were recruited at the time they arrived at the obstetrics department in CMCH. The informed consent was read thoroughly by the participants and was explicitly explained, with any doubts being resolved at that moment. Consent was provided by patients who agreed to participate and met the selection criteria.

A questionnaire was conducted to collect data related to medical records from the patients before and during pregnancy. Information regarding vaccination against SARS-CoV-2 status and history of COVID-19 was assessed, including the number of doses received, the type of vaccine used, the week of pregnancy in which each vaccine was administered, and medical history of COVID-19. Afterward, a blood sample of 2.5 mL from the mother was taken.

During childbirth, the gynecologist took a sample of 2.5 mL of blood directly from the umbilical cord. Data from the newborn were collected, including their name, sex, weight, and whether they needed to be transferred to the neonatal intensive care unit for handling. Before discharge and within the first 72 h after delivery, laboratory staff collected a 2.5 mL peripheral blood sample from the newborn by direct venipuncture.

The samples were centrifuged at 3500 revolutions for 15 min, and the plasma sample was kept at 4 °C in the laboratory area of the CMCH; later, they were sent to HCN to be processed using the LIASION^®^ SARS-CoV-2 S1/S2 kit, which consists of a chemiluminescence immunoassay to quantify the amounts of anti-S1 and anti-S2 specific IgG antibodies for SARS-CoV-2 with a sensitivity of 97.4% and specificity of 98.5%, considering a positive value greater than 15 AU/mL, indeterminate between 12 and 15 AU/mL, and negative value below 12 AU/mL.

The variables explored for the participants were their age, weight, and medical and obstetric history, such as hypertension, type 2 diabetes, obesity, SARS-CoV-2 history confirmed by a nasal swab test, gestational hypertension, preeclampsia, hypothyroidism, growth restriction, and gestational diabetes. The variables related to vaccination against SARS-CoV-2 were the number of doses, type of vaccine, and vaccination scheme received, classified as a homologous scheme if the vaccine applied was the same in all cases or a heterologous scheme if the type of vaccine administered differed between them. The variables related to the newborn were their gestational age and gender. The variables regarding the antibody titers were the three measurements taken of the anti-S1 and anti-S2 specific IgG antibodies for SARS-CoV-2, which, as mentioned, were taken from the pregnant woman before birth, umbilical cord during delivery, and newborn within the first 72 h after delivery.

The researchers reviewed the quality control and ensured patients’ anonymity in the database. The sample was determined by consecutive sampling. For the statistical analysis, the distribution of variables was explored, and normality was evaluated through the Kolmogorov–Smirnov test; antibody-related variables were normalized through logarithmic transformation. Frequencies, percentages, means, standard deviations, medians, and interquartile ranges were used as descriptive statistics. To compare the immune responses between distinct types of vaccines, we performed an ANOVA with Gabriel’s post hoc test using the homologous schedule population to avoid the bias of different immunological effects of heterologous vaccines in the same patient.

A generalized linear model was used to predict the changes in the levels of antibodies. The dependent variables were the levels of antibodies in maternal blood, umbilical cord blood, and newborn blood. The independent variables included maternal age, vaccination schedule, positive history of SARS-CoV-2, and the number of doses. A *p*-value less than 0.05 was considered statistically significant. The statistical program used for the analysis was IBM Statistics version 25 (SPSS).

## 3. Results

We recruited 104 pregnant women with a mean (SD) age of 29 (1.17) years. A total of 105 children were born in the study, two of which were twins, with 56 (53.3%) males. The median (IQR) gestational age was 38.6 (1.5) weeks, and the newborn’s mean (SD) birth weight was 3200 (424) gr. Sixty-one (58.7%) women reported a history of SARS-CoV-2 infection. During pregnancy, the most common diagnosis was gestational diabetes (n = 31, 29.8%), followed by gestational hypertension (n = 9, 8.7%) and hypothyroidism (n = 6, 5.8%). Table 1 describes the demographic and clinical characteristics of the population.

### 3.1. Vaccination Schemes

Regarding the number of shots received, eight (7.6%) only had one dose, forty-eight (45.7%) had two, forty-six (43.8%) had three, and two (1.9%) had four doses. Fifty-seven (54.8%) pregnant women received a homologous vaccination scheme while forty-seven (44.8%) received a heterologous scheme. Women received the last dose of the vaccination scheme at a median (IQR) of 19.2 (14) gestational weeks.

Homologous vaccination schemes were administered to 20 (34.5%) women with mRNA-1273, 20 (34.5%) with ChAdOx1-S, 16 (27.6%) with BNT162b2 mRNA, 1 (1.7%) with Ad5-nCoV, and 1 (1.7%) with Ad26.COV2. Regarding heterologous schemes, there were up to 16 different combinations reported, the most frequent being BNT162b2 mRNA/BNT162b2 mRNA/ChAdOx1-S (n = 16, 34.8%), followed by mRNA-1273/mRNA-1273/ChAdOx1-S (n = 11, 23.9%) (see Table 2).

### 3.2. Antibody Titers

All vaccinated women and their newborns reported seroconversion. The mean (SD) anti-S1/S2 antibody titers were 739.12 (2.37) AU/mL in maternal blood, 946.89 (56) AU/mL in umbilical cord blood, and 881.58 (2.69) AU/mL in newborns’ peripheral blood within the first 72 h of life. Women who received a homologous BNT162b mRNA regimen had a mean (SD) of 994.93 (3.08) AU/mL in maternal blood, 1316.43 (2.79) AU/mL in umbilical cord blood, and 1192.02 (3.55) AU/mL in newborn blood. In the homologous mRNA-1273 scheme, maternal blood exhibited 669.55 (2.38) AU/mL, umbilical cord blood exhibited 846.59 (2.36) AU/mL, and newborn blood exhibited 795.77 (2.39) AU/mL. The mean antibody titers in women receiving a homologous ChAdOx1-S scheme were 385.37 (3.0) in maternal blood, 461.68 (2.95) AU/mL in umbilical cord blood, and 424.68 (2.57) in newborn blood. From this group of homologous vaccination, women vaccinated with BNT162b mRNA had the highest antibody titers in the maternal, umbilical cord, and newborn blood (*p* = 0.039, *p* = 0.016, and *p* = 0.02, respectively) (See Table 3).

Subjects with homologous regimens were divided according to SARS-CoV-2 infection history. In women with a positive history of infection and homologous regimen, the BNT162b mRNA scheme had the significantly highest mean (SD) of anti-S1/S2 antibody titers in the umbilical cord and newborn blood (1759.31 (1.73) AU/mL and 1701.1 (1.7) AU/mL, respectively) compared with mRNA-1273 (929.79 (2.48) AU/mL and 904.15 (2.46) AU/mL) and ChAdOx1-S (473.32 (3.44) AU/mL 438.46 (2.86) AU/mL) (*p* = 0.034 and *p* = 0.013, respectively). There was no significant difference in women with negative SARS-CoV-2 infection history. In Table 4, the mean antibody titers are reported by vaccine and history of SARS-CoV-2 infection in the homologous scheme group. 

We created a generalized linear model where the dependent variables were the log10 antibody titers in the maternal, umbilical cord, and newborn blood. The independent variables were the maternal age, homologous or heterologous scheme, positive history of SARS-CoV-2, and the number of doses of vaccines received. Regarding antibody titers in maternal blood, the administration of at least one dose of vaccine had a positive effect (one dose β = −1.1, (*p* = 0.002), two doses β = −1.1, (*p* = 0.001), and three doses β = −1.1, (*p* = 0.004)). There was no significant effect on maternal age and the type of vaccination regimen (heterologous or homologous). In newborn blood, antibody titers were positively affected by the one-dose (β = −0.717, *p* = 0.044) and two-dose (β = −0.788, *p* = 0.015) vaccine regimens. However, in umbilical cord blood, only a two-dose regimen had a significantly positive effect on the antibody titers (β = −0.684, *p* = 0.026). Table 5 shows the generalized linear model where the antibody titers are the dependent variable.

## 4. Discussion

This study showed the transplacental transfer of antibodies against SARS-CoV-2 from mothers receiving one to four doses of SARS-CoV-2 vaccination from BNT162b2, ChAdOx1-S, Ad5-nCoV, Coronavac, Ad26.COV2, and Gam-COVID-Vac.

The median time at which the last dose was administered was during the 19th week of pregnancy. In the homologous group, BNT162b2 showed the highest concentration of IgG vs. Spike 1 and 2.

In the present study, the whole sample had successfully transferred antibodies to their newborns before birth, as shown by their presence in the umbilical cord and peripheral blood samples. The transfer of transplacental antibodies against SARS-CoV-2 has been demonstrated in different studies [25,26,27,28,29,30,31]. As seen in the study by Kugelman et al., which focused on 129 pregnant women vaccinated with at least two doses of BNT162b2 and no history of COVID-19, the total sample had IgG antibodies in the maternal blood and the umbilical cord blood [28].

Nir O et al. [27] described the transmission of antibodies in 64 patients vaccinated with at least two doses of BNT162b2 compared with 11 unvaccinated patients with a history of COVID-19 during pregnancy, finding significant differences in the numbers of antibodies between the two groups, with the transfer being higher in vaccinated mothers. In our study, pregnant women with a history of COVID-19 and SARS-CoV-2 vaccination presented a higher number of antibodies in both the umbilical cord blood and peripheral blood of the newborn (*p* = 0.034 and *p* = 0.013, respectively).

Our study observed an increase in the number of antibodies in umbilical cord blood compared with the number of antibodies seen in maternal blood (mean 946.89 Ur/L compared to 739.12 Ur/L), similar to what was observed in the Kugelman et al. cohort [30], where they reported a mean of 1185.2 AU/mL in maternal blood and a mean of 3315.7 AU/mL in umbilical cord blood. This could be explained by the reduced blood volume in the umbilical cord, increased transfer of antibodies during labor, or active and passive transplacental transmission [26,27,28,29,30].

Most studies have compared the application of the second dose in homologous schemes. Mithal L et al. [32] collected a series of cases involving 27 pregnant women; 64% received BNT162b2, 18% received mRNA-1273, and 4% received another type of vaccination without making comparisons between the different schemes because the sample of patients was small. Sourouni M et al. [33], in their study of 70 vaccinated pregnant women, 89% with BNT16b2 and 10% with mRNA-1273, showed that there was no correlation between antibody levels and the week of gestation at which vaccination was performed, the time interval between birth and vaccination, maternal age, or the mother’s body mass index. Like Sourouni M et al., we found, in our study using the generalized linear model, that significance was seen in the number of vaccine doses administered rather than the maternal age, type of vaccine regimen, and history of positivity for COVID-19. It is important to add that some patients received the vaccine in the first weeks of pregnancy, and they were also able to transfer antibodies at birth.

Prahl et al. conducted a study where 20 pregnant women were included to measure the impact of mRNA vaccination against SARS-CoV-2. They concluded that, although mRNA and spike proteins derived from the vaccine’s components were absent in blood, IgG and neutralizing antibodies were present in the cord (88%) and newborn (81%) blood. The absence of antibodies in cord and newborn blood was derived from the lack of antibodies in the mothers. Similarly, our study affirms this conclusion, because we found that anti-SARS-CoV-2 IgG antibodies were present in maternal, cord, and newborn blood, even though we did not measure the levels of vaccine components and neutralizing antibodies in the blood. Additionally, our findings were seen in all participants, despite the differences in the vaccination schemes and the number of vaccines administered [34].

The novelty of our study is that we analyzed and compared different types of vaccines using homologous and heterologous schemes during pregnancy. In addition, we also analyzed patients that received up to four doses, which allowed us to compare the different vaccination schemes. Previously published studies showed results regarding BNT162b2, mRNA1273, and Ad26.COV2.S [35,36]. Many countries in the world have limited access to these types of vaccines. To the best of our knowledge, this is a real-world study that analyzes other types of vaccines that have not been previously compared, such as ChAdOx1-S, CoronaVac, and Gam-COVID-Vac, as well as 47 heterologous regimens. This is what the population has access to, and we surprisingly found that no matter the type of vaccine, if pregnant women received at least one dose, the antibodies would be present in the newborn. A previous systematic review and meta-analysis demonstrated that only 27.5% of pregnant women have been vaccinated against COVID-19. Mistrust in the government, fears about safety, and side effects of COVID-19 are related to declining vaccination [37]. Another study examined the predominant negative opinions related to COVID-19 vaccination through public Twitter posts, reporting skepticism about COVID-19 vaccines’ effectiveness against emerging variants and criticism of vaccine mandates and regulations [38]. The results of our study encourage pregnant women to get vaccinated with any available vaccination scheme.

The main study limitation lies in the sample size, which is considered small, the same as other studies with similar or smaller sample sizes. We did not have a control group to compare the data. For future research, a cohort study should be developed to determine the duration for which these antibodies persist in the newborn’s blood after delivery and determine how it varies between vaccination schemes. Breast milk antibodies may be part of the serum IgG present in the first 72 h after birth; we also think that further studies must be conducted to measure the immune response in breast milk from mothers vaccinated with different schemes.

## 5. Conclusions

This study determined the presence of anti-S1 and anti-S2 specific IgG antibodies for SARS-CoV-2 in the mother, the umbilical cord, and the newborns’ blood within the first 72 h after birth. Though it confirms the transfer of the antibodies from the mother to the newborn in vaccinated women, we found no significant difference between homologous and heterologous vaccination schemes.

Transplacental transfer occurred since the application of the first vaccine, which increased with each dose; this effect was independent of the vaccination scheme. Regardless of the trimester in which the women received their last dose (first or third trimester), all of them were able to transfer antibodies to the newborn.

## Figures and Tables

**Table 1:** Demographic and Clinical Characteristics.

Maternal Characteristics	n = 104
Age; mean (SD), years	29 (1.17)
History of SARS-CoV-2, N, (%)	
Total positive	61 (58.7)
Positive during pregnancy *	39 (63.9)
Days after conception; median (IQR)	114 (91)
Positive before pregnancy	22 (36.1)
Days before conception	152 (342)
Negative	43 (41.3)
Maternal disease, n, (%)	
Gestational hypertension	9 (8.7)
Preeclampsia without severity data	2 (1.9)
Preeclampsia with severity data	1 (1)
Gestational diabetes	31 (29.8)
Hypothyroidism	6 (5.8)
Newborn	N = 105
Gestational age at birth, median (IQR), weeks	38.6 (1.5)
Male (%)	56 (53.3)
Newborn weight at birth, mean (SD), g	3200 (424.1)

* One patient was positive for COVID-19 during labor.

**Table 2:** Description of the vaccination schemes.

Number of Vaccines	n (%)
1 vaccine	8 (7.6)
2 vaccines	48 (45.7)
3 vaccines	46 (43.8)
4 vaccines	2 (1.9)
Vaccination schedule	
Homologous	57 (54.8)
Heterologous	47 (44.8)
Homologous schedule	
BNT162b2 mRNA	16 (27.6)
mRNA-1273	20 (34.5)
ChAdOx1-S	20 (34.5)
Ad5-nCoV	1 (1.7)
Ad26.COV2	1 (1.7)
Heterologous schedule	
BNT162b2 mRNA/BNT162b2 mRNA/ChAdOx1-S	16 (34.8)
mRNA-1273/mRNA-1273/ChAdOx1-S	11 (23.9)
mRNA-1273/BNT162b2 mRNA	3 (6.5)
Ad5-nCoV/BNT162b2 mRNA	2 (4.3)
mRNA-1273/mRNA-1273/Ad5-nCoV	2 (4.3)
ChAdOx1-S/ChAdOx1-S/BNT162b2 mRNA	2 (4.3)
Other ^a^	10 (22)

^a^ Represent 10 different types of schedule (BNT162b2 mRNA/BNT162b2 mRNA/Gam-COVID-Vac, mRNA-1273/ChAdOx1-S, Ad5-nCoV/ChAdOx1-S, BNT162b2 mRNA/ChAdOx1-S, Coronavac/Coronavac/ChAdOx1-S, Ad5-nCoV/mRNA-1273/mRNA-1273, BNT162b2 mRNA/BNT162b2 mRNA/ChAdOx1-S/BNT162b2 mRNA, mRNA-1273/mRNA-1273/BNT162b2 mRNA, and Ad5-nCoV/mRNA-1273/mRNA-1273/BNT162b2 mRNA, Ad5-nCoV/mRNA-1273).

**Table 3:** Anti-Spike 1 and 2 IgG Comparison between the most frequent homologous vaccination schedules.

Antibodies	BNT162b2 mRNA	mRNA-1273,	ChAdOx1-S,	*p*-Value
Maternal sample, mean (SD), AU/mL	994.93 (3.08) ^a^	669.55 (2.38) ^b^	385.37 (3.00) ^b^	0.039
Umbilical cord sample, mean (SD), AU/mL	1316.43 (2.79) ^a^	846.59 (2.36) ^b^	461.68 (2.95) ^b^	0.016
Newborn sample, mean (SD), AU/mL	1192.02 (3.55) ^a^	795.77 (2.39) ^b^	424.68 (2.57) ^b^	0.02

^a,b^ ANOVA with Gabriel’s post hoc test. Log10 transformation was used for the anti-S1/S2 antibody titers of maternal, umbilical cord, and newborn blood.

**Table 4:** Comparison between homologous vaccination schedules with maternal history of SARS-CoV-2.

Antibodies	BNT162b2 mRNA	mRNA-1273,	ChAdOx1-S,	*p*-Value
Positive history of SARS-CoV-2				
Maternal sample, mean (SD), AU/mL	1062.13 (2.24)	727.29 (2.42)	415.82 (3.39)	0.162
Umbilical cord sample, mean (SD), AU/mL	1759.31 (1.73) ^a^	929.79 (2.48) ^a^	472.32 (3.44) ^b^	0.034
Newborn sample, mean (SD), AU/mL	1701.10 (1.70) ^a^	904.15 (2.46) ^a^	438.46(±2.86) ^b^	0.013
Negative history of SARS-CoV-2				
Maternal sample, mean (SD), AU/mL	931.98 (4.2)	601.99 (2.45)	338.27 (2.54)	0.263
Umbilical cord sample, mean (SD), AU/mL	985.04 (3.81)	750.46 (2.32)	443.99 (2.34)	0.365
Newborn sample, mean (SD), AU/mL	835.30 (5.50)	675.30 (2.38)	402.05 (2.22)	0.514

^a,b^ ANOVA with Gabriel’s post hoc test. Log10 transformation was used for the anti-S1/S2 antibody titers of maternal, umbilical cord, and newborn blood.

**Table 5:** Prediction of the numbers of anti-S1 and anti-S2 specific IgG antibodies for SARS-CoV-2.

	β	SE	Significance	95% CI	η^2^
Maternal antibodies intersection	3.778	0.455	<0.001	2.878 to 4.68	0.418
Maternal age	0.002	0.009	0.823	−0.015 to 0.019	0.001
Homologous or heterologous	0.001	0.113	0.99	−0.223 to 0.226	<0.001
History of COVID-19	0.031	0.087	0.721	−0.141 to 0.203	0.001
1 vaccine	−1.1	0.35	0.002	−1.795 to −0.405	0.093
2 vaccines	−1.1	0.317	0.001	−1.729 to −0.405	0.111
3 vaccines	−0.905	0.303	0.004	−1.506 to −0.305	0.085
4 vaccines					
Umbilical cord antibodies intersection	3.348	0.435	<0.001	2.485 to 4.21	0.382
Maternal age	0.004	0.008	0.642	−0.013 to 0.02	0.002
Homologous or heterologous	0.031	0.108	0.777	−0.184 to 0.245	0.001
History of COVID-19	0.075	0.083	0.366	−0.089 to 0.24	0.009
1 vaccine	−0.61	0.334	0.071	−1.273 to 0.054	0.033
2 vaccines	−0.684	0.303	0.026	−1.285 to −0.083	0.05
3 vaccines	−0.487	0.289	0.096	−1.061 to 0.88	0.029
4 vaccines					
Newborn antibodies intersection	3.341	0.457	<0.001	2.43 to 4.24	0.358
Maternal age	0.006	0.009	0.492	−0.011 to 0.023	0.005
Homologous or heterologous	0.031	0.114	0.784	−0.194 to 0.257	0.001
History of COVID-19	0.091	0.087	0.300	−0.082 to 0.263	0.011
1 vaccine	−0.717	0.351	0.044	−1.414 to −0.02	0.042
2 vaccines	−0.788	0.318	0.015	−1.419 to −0.157	0.06
3 vaccines	−5.81	3.04	0.059	−1.18 to 2.20	0.037
4 vaccines					

Log10 transformation was used for the anti-S1/S2 antibody titers of maternal, umbilical cord, and newborn blood.

## Data Availability

Data are available upon reasonable request to corresponding author.
